# Open-Sourced CIViC Annotation Pipeline to Identify and Annotate Clinically Relevant Variants Using Single-Molecule Molecular Inversion Probes

**DOI:** 10.1200/CCI.19.00077

**Published:** 2019-10-16

**Authors:** Erica K. Barnell, Adam Waalkes, Matt C. Mosior, Kelsi Penewit, Kelsy C. Cotto, Arpad M. Danos, Lana M. Sheta, Katie M. Campbell, Kilannin Krysiak, Damian Rieke, Nicholas C. Spies, Zachary L. Skidmore, Colin C. Pritchard, Todd A. Fehniger, Ravindra Uppaluri, Ramaswamy Govindan, Malachi Griffith, Stephen J. Salipante, Obi L. Griffith

**Affiliations:** ^1^ Washington University School of Medicine, St Louis, MO; ^2^ University of Washington, Seattle, WA; ^3^ University of California, Los Angeles, Los Angeles, CA; ^4^ Charité Unviersitätsmedizin Berlin, Berlin, Germany; ^5^ Brigham and Women’s Hospital and Dana-Farber Cancer Institute, Boston, MA

## Abstract

**PURPOSE:**

Clinical targeted sequencing panels are important for identifying actionable variants for patients with cancer; however, existing approaches do not provide transparent and rationally designed clinical panels to accommodate the rapidly growing knowledge within oncology.

**MATERIALS AND METHODS:**

We used the Clinical Interpretations of Variants in Cancer (CIViC) database to develop an Open-Sourced CIViC Annotation Pipeline (OpenCAP). OpenCAP provides methods to identify variants within the CIViC database, build probes for variant capture, use probes on prospective samples, and link somatic variants to CIViC clinical relevance statements. OpenCAP was tested using a single-molecule molecular inversion probe (smMIP) capture design on 27 cancer samples from 5 tumor types. In total, 2,027 smMIPs were designed to target 111 eligible CIViC variants (61.5 kb of genomic space).

**RESULTS:**

When compared with orthogonal sequencing, CIViC smMIP sequencing demonstrated a 95% sensitivity for variant detection (n = 61 of 64 variants). Variant allele frequencies for variants identified on both sequencing platforms were highly concordant (Pearson’s *r* = 0.885; n = 61 variants). Moreover, for individuals with paired tumor and normal samples (n = 12), 182 clinically relevant variants missed by orthogonal sequencing were discovered by CIViC smMIP sequencing.

**CONCLUSION:**

The OpenCAP design paradigm demonstrates the utility of an open-source and open-access database built on attendant community contributions with peer-reviewed interpretations. Use of a public repository for variant identification, probe development, and variant interpretation provides a transparent approach to build dynamic next-generation sequencing–based oncology panels.

## INTRODUCTION

Despite recognition that genomics plays an important role in tumor prognosis, diagnosis, and treatment, scaling genetic analysis for routine analysis of most tumor specimens has been unattainable.^1,2^ Barriers preventing widespread incorporation of genomic analysis into treatment protocols include costs associated with genomic sequencing and analysis,^3^ computational limitations preventing timely identification of relevant variants,^3^ and rapidly evolving knowledge of the clinical actionability of variants.^4^ Technologic improvements in sequencing and data analysis continue to reduce these first 2 limitations; however, less progress has been made in integrating dynamic genomic annotation into clinical workflows. More than 22% of oncologists have acknowledged limited confidence in their own understanding of how genomic knowledge applies to patients’ treatment, and 18% reported testing patients’ genetics infrequently.^5^ In the face of exponential growth in clinically relevant genomic findings, driven by precision oncology efforts, there will likely be increased inability for physicians to command the most current information, resulting in increasing delay between academic discovery and clinical utility. This information gap has been described as the interpretation bottleneck.^4-6^

Alleviating the interpretation bottleneck will require codevelopment of targeted sequencing panels, bioinformatic tools, and variant knowledgebases that effectively elucidate and annotate clinically actionable variants from sequencing data.^7,8^ These requirements each raise separate challenges. With regard to targeted panel development, commercial and academic pancancer clinical gene capture panels have now become commonplace, with at least 2 obtaining US Food and Drug Administration approval (FoundationONE CDx^9^ [Foundation Medicine, Cambridge, MA] and Memorial Sloan Kettering-Integrated Mutation Profiling of Actionable Cancer Targets^10^ [Memorial Sloan Kettering Cancer Center, New York, NY]). Even so, few panels indicate how genomic loci are selected for panel inclusion (Data Supplement), and none have proposed a sustainable or scalable mechanism to allow for panel evolution over time in response to knowledge advances in molecular oncology. With regard to bioinformatic tool development, the OncoPaD^11^ portal provides one of the only methods to create rational designed panels by linking clinically relevant variants to genomic loci on the basis of a cohort of tumor samples; however, this database is not directly linked to actively updated clinical interpretations with detailed underlying evidence. The final challenge of building knowledgebases for variant interpretation perhaps poses even greater and more persistent challenges. Commercial entities typically rely on the manual curation and organization of research findings into structured databases, which are expensive to create and maintain, forcing companies to limit public access or to charge for use. The resulting lack of transparency creates inefficiencies in the field through unnecessary replication of curation effort and suboptimal communication with clinicians, ultimately hindering development of effective patient treatment plans. Separately, governmental and academic institutions have developed variant interpretation resources, such as the Catalogue of Somatic Mutations in Cancer,^12^ ClinVar,^13^ and cBioPortal,^14,15^ that have drastically improved research efforts and academic discovery; however, these resources do not have well-supported (evidence-based) clinical relevance summaries for cancer variants that can be easily accessed and used by physicians. Several resources provide detailed clinical interpretation of cancer variants (eg, OncoKB,^16^ JAX Clinical Knowledgebase,^17^ and others), but these databases are either limited by license restrictions or closed curation models.

**Context**

**Key Objective**
Development of clinical genomics pipelines and associated analytical software is needed to meet the growing needs of oncologists for cancer diagnosis and treatment.
**Knowledge Generated**
Here we describe methods for using the Clinical Interpretations of Variants in Cancer (CIViC) database to develop the Open-Sourced CIViC Annotation Pipeline (OpenCAP). This resource first describes methods for variant capture and subsequently provides tools for variant annotation. Using OpenCAP, we demonstrated applicability through development of a single-molecule molecular inversion probe capture panel, which was validated against whole-exome sequencing.
**Relevance**
Maintenance and continuous improvement of the OpenCAP software will help to serve the needs of researchers and physicians who are using precision oncology to guide treatment of their patients.

**FIG 1.**
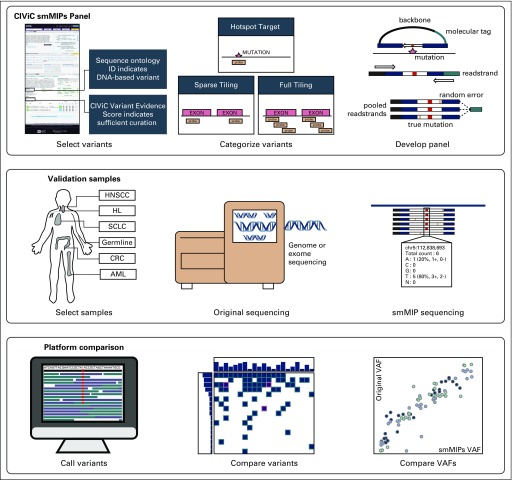
Methods for Clinical Interpretations of Variants in Cancer (CIViC) single-molecule molecular inversion probe (smMIP) development and validation using the Open-Sourced CIViC Annotation Pipeline (OpenCAP). The first series describes CIViC smMIP development. Variants were selected using sequence ontology identification numbers (IDs) and the CIViC Variant Evidence Score. Subsequently, eligible variants were categorized based on length, and smMIP reagents were designed to target regions of interest. The second series describes sample selection and sequencing methods. In total, there were 22 tumor samples derived from 5 tumor subtypes. Of these 27 samples, 15 had tumor and paired normal samples, and 7 were tumor-only samples. The third series shows the analysis used to validate the CIViC smMIP design. Variants were called using the pipeline described in Materials and Methods, and accuracy was attained by comparing variants observed on original sequencing to variants observed using the CIViC smMIP capture panel. Variant allele frequencies (VAFs) across both platforms were also compared. AML, acute myeloid leukemia; CRC, colorectal cancer; HL, Hodgkin lymphoma; HNSCC, head and neck squamous cell carcinoma; SCLC, small-cell lung cancer.

To address these limitations, we developed a method to identify, capture, and annotate variants using the Clinical Interpretation of Variants in Cancer (CIViC) database.^18^ The CIViC database is a freely accessible (public domain content), publicly curated, expert-moderated repository of therapeutic, prognostic, predisposing, and diagnostic information in precision oncology.^19^ The database provides a powerful platform for panel development and variant annotation for the following reasons: each variant within CIViC is described by clinical relevance summaries linked to medical literature; the history of curation within CIViC is stored and publicly available to all users; and CIViC has an open-source, open-access applied programming interface (API) for external query. Using the CIViC database and API, we developed the Open-Sourced CIViC Annotation Pipeline (OpenCAP) for creating custom capture panels, executing capture panel sequencing on prospective samples, identifying variants from sequencing data, and annotating variants for clinical relevance.^20^ An exemplary clinical capture panel was created using OpenCAP to demonstrate utility. Specifically, variants within the CIViC database were identified based on clinical relevance, and single-molecule molecular inversion probes (smMIPs) were designed to target variants of interest. This panel was used on cancer samples to evaluate design, and identified somatic variants were compared with orthogonal sequencing. Variants identified via smMIP capture were linked back to the CIViC database for clinical annotation (Fig 1). Ultimately, this method could be used to rapidly and efficiently link variants to clinical relevance summaries, enabling the development of custom capture panels for a variety of clinical and research scenarios.

## MATERIALS AND METHODS

### Development of Operating Procedure for OpenCAP

OpenCAP was built to guide users through the development of a custom capture panel linked to CIViC clinical relevance summaries.^20^ OpenCAP consists of 5 sections, each with examples and user tutorials. The first section describes CIViC and directs users through the CIViC Web interface. The next section describes methods for building a custom capture panel, which includes identifying pertinent variants within the CIViC database and targeting those variants with probes using curated genomic coordinates. Subsequently, OpenCAP gives a high-level overview of the massively parallel sequencing pipeline, which includes brief summaries for sample procurement, nucleic acid generation, library preparation, and high-throughput sequencing. The final sections describe identifying variants from raw sequencing data and annotating those variants for clinical relevance.

### Determining Eligible CIViC Variants for smMIP Capture

Variants in CIViC were filtered using their Variant Evidence Score (required > 20 points) and sequence ontology identification numbers (SOIDs; must be DNA based; Appendix). Variants were also filtered if all evidence supported only germline clinical relevance, evidence was directly conflicting, or a majority of evidence in a container variant (eg, MUTATION) pointed to a hotspot that was already being covered. The remaining variants were eligible for the CIViC smMIP capture panel.

### Designing smMIPs for the CIViC Capture Reagents

Variants were further categorized by length. If the variant length was < 250 base pairs, the variant was eligible for hotspot targeting. If the variant was > 250 base pairs, the variant required either sparse or full tiling of the protein coding exons (Appendix). For all variants, smMIPs were designed and synthesized as previously described^23^ with the single alteration that the “-double_tile_strands_separately”^24^ flag was used with the MIPgen tool to separately capture each strand of DNA surrounding the target.

### Rescue and Annotation of Clinically Relevant Variants

Variants called using the CIViC smMIP capture panel were compared with variants called using original sequencing for samples that had matched tumor and normal sequencing. All genomic loci were manually reviewed^23^ using both the smMIP aligned Binary Alignment Map (BAM) files and the original aligned BAM files. Variants only identified using smMIP sequencing were grouped into the following 4 categories: germline polymorphism, pipeline artifact (low variant support or poor mapping), variant support on smMIP sequencing but no support on original sequencing, or variant support on both smMIP sequencing and original sequencing. For variants that showed support on smMIP sequencing but no variant support on original sequencing, the binomial probability was used to assess whether ≤ 3 variant-supporting reads would be detected with 95% confidence using the original coverage and the observed smMIP variant allele frequency (VAF). The accession number for the first release of the Database of Genotypes and Phenotypes study was phs001890.v1.p1, and the accession number for first release of the Sequence Read Archive was PRJNA529857.

## RESULTS

### Identification of Eligible CIViC Variants for smMIP Targeting

**FIG 2.**
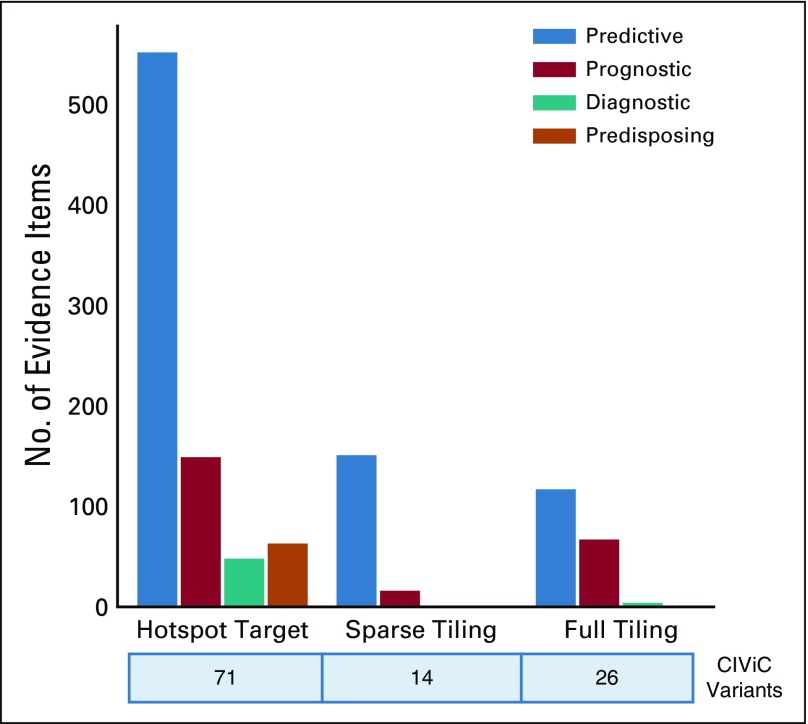
Regions targeted by the Clinical Interpretations of Variants in Cancer (CIViC) single-molecule molecular inversion probes (smMIPs) are, by design, supported by extensive clinical relevance according to the CIViC database. Variants that were eligible for CIViC smMIP development were divided into various coverage methods based on sequence ontology identification number and length. The bar graph shows the total number of evidence items used for each of the groups parsed by the evidence type.

At the time of the CIViC smMIP capture panel design, there were 988 variants from 275 genes within the CIViC database with at least 1 evidence item. After filtering based on the Variant Evidence Score and the SOID (Appendix, Data Supplement), smMIPs were designed to cover all eligible CIViC variants. A set of 2,097 probes was developed and tested on control samples. Of these, 70 probes showed poor capture efficiency and were eliminated from the panel. Removal of the underperforming probes affected 32 variants across 16 genes. The final capture reagent targeted 111 CIViC variants spanning approximately 61.5 kb of genomic space (Data Supplement). When compared with other pancancer panels, the CIViC capture panel showed high overlap with previously defined clinical variants. For example, the CIViC smMIP capture panel covered 10 of the 13 well-defined variants on FoundationOne CDx (*EGFR*: exon 19, L858R, and T790M; *BRAF*: V600E/K; *ERBB2* amplification; *KRAS* G12/13; *BRCA1*; and *BRCA2*).^24^ The 3 variants on FoundationOne CDx that were not originally covered by the smMIPs panel (*KRAS* wild type, *NRAS* wild type, and *ALK* rearrangements) have all since attained a Variant Evidence Score that would be sufficient for inclusion in a panel built today. Of the 111 targeted variants, 71 required hotspot targeting, 14 variants required sparse exon tiling, and 26 required full exon tiling. The 111 variants covered by the CIViC smMIP capture panel were based on 1,168 clinically relevant evidence items, whereby 820 evidence items (70%) predicted response to a therapeutic, 232 (20%) detailed prognostic information, 52 (4%) indicated diagnostic information, and 64 (6%) supported predisposition to cancer (Fig 2).

### Tumor Samples Used to Validate CIViC smMIP Design

Samples used to validate the CIViC smMIP capture panel design were derived from 5 different cancer genomic studies (Data Supplement). Tumor and paired normal samples were obtained from 5 individuals with head and neck squamous cell carcinoma (HNSCC), 9 individuals with small-cell lung cancer,^25^ and 1 individual with Hodgkin lymphoma (HL). Tumor-only samples were obtained from 1 individual with HL, 1 individual with acute myeloid leukemia,^26^ and 5 individuals with colorectal cancer (CRC). In total, 37 samples were evaluated from 22 individuals. Samples from the CRC cohort were formalin-fixed paraffin-embedded, and all other samples were fresh frozen tissue.

Each of the 22 individuals had previously undergone whole-exome or whole-genome sequencing, somatic variant calling, and somatic variant refinement via manual review (Appendix). Considering original sequencing, there were 12,602 putative somatic variants called for these 22 samples. The average variant burden was 573 variants per sample, with a range of 2-3,900 variants per sample. Variant coordinates from these samples were compared with the genomic region covered by the CIViC smMIP capture panel to determine potential validating variants. In total, there were 84 variants identified via original sequencing that overlapped with the CIViC smMIP capture panel (Data Supplement).

### smMIP Sequencing and Data Analysis

#### Initial quality check.

**FIG 3.**
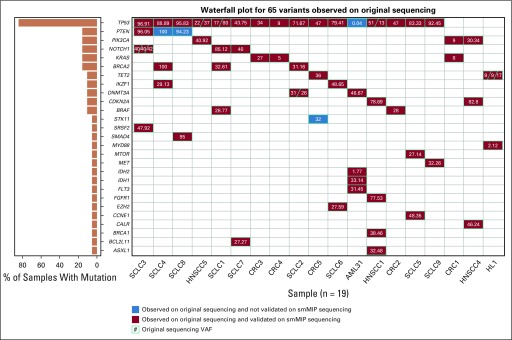
Waterfall plot showing extensive overlap between variants observed using original exome or whole-genome sequencing with variants observed using Clinical Interpretations of Variants in Cancer (CIViC) single-molecule molecular inversion probe (smMIP) sequencing. Each column represents a sample that had original exome or whole-genome sequencing with subsequent orthogonal validation using the CIViC smMIP sequencing. Rows represent mutated genes across all samples. Numbers within each box represent the variant allele frequency (VAF) observed on original exome or whole-genome sequencing. Red boxes indicate that a variant was observed by CIViC smMIPs and validated with original exome or whole-genome sequencing. Blue boxes indicate that the variant was observed on original exome or whole-genome sequencing but not identified via the CIViC smMIP capture panel. The left panel indicates the number of samples containing a mutation in the indicated gene. AML, acute myeloid leukemia; CRC, colorectal cancer; HL, Hodgkin lymphoma; HNSCC, head and neck squamous cell carcinoma; SCLC, small-cell lung cancer.

The average number of tags captured for all samples was 5.4 million (standard deviation, 3.3 million tags). One HNSCC normal sample failed smMIP capture, 2 HNSCC tumor samples had significantly fewer reads than the rest (ie, > 1 standard deviation), and 1 HL tumor sample had reduced tag complexity relative to the rest (ie, < 600,000 unique captured smMIPs). Sequencing failure for these 4 samples was attributable to poor template quality or quantity and not attributable to the capture reagents. All other samples passed sequencing quality checks. After quality check, 31 samples derived from 19 individuals were eligible for reagent validation. These samples had 65 variants derived from orthogonal sequencing that had overlap with the CIViC smMIP coverage (Fig 3). The average consensus read depth for these 65 variants was 2,942 reads (standard deviation, 4,697 reads).

#### Accuracy of CIViC smMIP variant identification compared with exome or genome variant identification.

Of the 65 variants identified on exome sequencing, all but 4 were also identified using CIViC smMIP sequencing (Fig 3). One variant was missed as a result of lack of adequate coverage, 2 variants were missed as a result of low-performing probes, and 1 variant was retrospectively considered ineligible as a result of smMIP design (Appendix). After removing this variant from the list of eligible variants, the CIVIC smMIP capture sequencing attained a 95% sensitivity for variant detection (n = 64 variants).

#### VAF correlation between CIViC smMIP sequencing and exome or genome sequencing.

**FIG 4.**
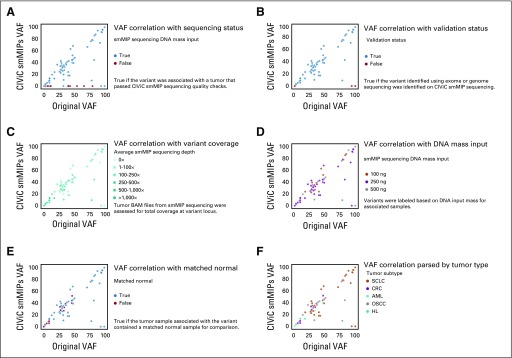
Variant allele frequencies (VAFs) observed using original exome or whole-genome sequencing compared with VAFs observed using Clinical Interpretations of Variants in Cancer (CIViC) single-molecule molecular inversion probe (smMIP) sequencing. (A) Correlation of VAF with original sequencing parsed by sequencing status (ie, passed sequencing if total sequencing counts were > 1 standard deviation from the mean and tag complexity was > 600,000 unique captured smMIPs). (B) Correlation of VAF with validation status (ie, true if the variant identified using exome or genome sequencing was identified on CIViC smMIP sequencing). (C) Correlation of VAF parsed by coverage at variant loci. (D) Correlation of VAF parsed by DNA mass input for library construction. (E) Correlation of VAF parsed by presence or absence of matched normal tissue. (F) Correlation of VAF parsed by tumor type. AML, acute myeloid leukemia; CRC, colorectal cancer; HL, Hodgkin lymphoma; OSCC, oral squamous cell carcinoma; SCLC, small-cell lung cancer.

VAFs obtained via original sequencing were compared with the VAF obtained using the CIViC smMIPs. To compare VAF quantitation across platforms, the 19 variants obtained from samples that failed the CIViC smMIP sequencing quality check were eliminated (Fig 4A). Subsequently, we eliminated the 4 variants that were not validated using the CIViC smMIP reagents (Fig 4B). When comparing original VAFs to CIViC smMIP VAFs, Pearson correlation for the remaining 61 variants was 0.885. There were several variants whereby the VAF observed by the CIViC smMIP sequencing was lower than that observed by the original sequencing. These outliers were not associated with tumor type, sequencing mass input, average coverage, presence of matched normal, or sample type (Figs 4C to 4F).

### Analysis of Variants Only Identified Using CIViC smMIP Sequencing

**FIG 5.**
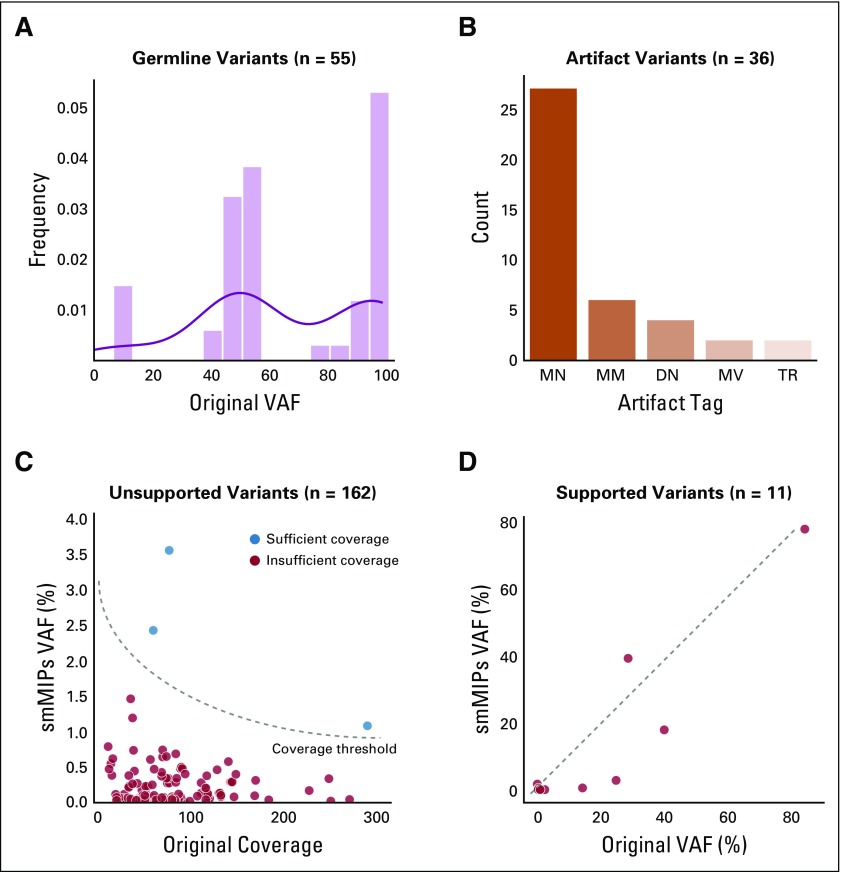
Analysis of variants rescued by Clinical Interpretations of Variants in Cancer (CIViC) single-molecule molecular inversion probe (smMIP) sequencing for samples with both tumor and matched normal. There were 217 variants called as somatic by CIViC smMIP sequencing that were not identified by the original sequencing. All variants were manually reviewed using both CIViC smMIP sequencing data and original sequencing data. (A) During manual review, 55 variants were identified as germline. A histogram shows that the distribution of the smMIP variant allele frequencies (VAFs) for these germline variants was observed at 50% and 100% VAF, indicating heterozygosity and homozygosity, respectively. (B) An additional 36 variants were identified as sequencing artifacts. Most artifacts were either mononucleotide repeats (MN), dinucleotide repeats (DN), or tandem repeats (TR). Other artifacts include multiple mismatches (MM) or multiple variants (MV). (C) During manual review, 162 variants did not show any support in the original sequencing data. Most unsupported variants did not have sufficient coverage to be detected based on a binomial probability of ≤ 3 variant-supporting reads (see Materials and Methods). (D) The remaining 11 variants had variant support in original sequencing but were not called as somatic in final original annotation. The scatter plot shows correlation between original VAF and CIViC smMIP VAF for these variants.

Using samples that had sequencing data for both tumor and matched normal (n = 12 samples), we evaluated whether the targeted CIViC smMIP sequencing could identify clinically relevant variants that had not been observed by the original sequencing. There were 273 variants recovered by CIViC smMIP sequencing that were not identified using original sequencing. After manually reviewing these variants within the original exome or genome alignments, 55 variants (20.1%) were identified as germline mutations. smMIP sequencing VAF distribution at 50% and 100% further supported that these variants were germline polymorphisms (Fig 5A). An additional 36 variants (13.2%) were thought to be caused by pipeline artifacts and attributable to assumptions underlying automated callers or alignment problems. The majority of these artifacts were associated with nucleotide repeats in the reference sequence (Fig 5B). There were 171 variants (62.6%) called as somatic using CIViC smMIPs that did not have any variant support on the original sequencing. For these variants, we calculated the binomial probability that ≤ 3 reads would support the variant given the original coverage (number of chances to get a variant supporting read) and the observed smMIP VAF (likelihood that a read would show variant support). If the binomial probability of ≤ 3 variant-supporting reads was > 95%, then it was considered statistically unlikely that a variant would be called using original sequencing data. Using this calculation, 162 variants (94.7%) showed insufficient coverage in the original sequencing for detection (Fig 5C). Finally, 11 variants (4.2%) were not called as somatic on original sequencing but did show some variant support in those original sequencing data. The VAFs observed on original sequencing data were strongly correlated with the VAFs observed using CIViC smMIP sequencing (Pearson’s *r* = 0.92; Fig 5D). Reviewing manual review files from the original sequencing, we observed that 6 of these variants failed manual review as a result of low VAF, 4 variants had not been called by automated somatic variant callers, and 1 variant failed manual review as a result of a perceived sequencing artifact. In summary, there were 182 potentially clinically relevant somatic variants missed by original sequencing, primarily as a result of insufficient coverage, that contained CIViC variant annotations.

### Annotation of CIViC smMIP Capture Panel Somatic Variants Using OpenCAP

Using the OpenCAP annotation software, we developed clinical interpretation reports for all variants observed using the CIViC smMIP capture panel. In total, there were 1,340 variants observed across the 19 samples that passed smMIP sequencing. Of the 1,340 variants observed, 127 had direct matches (chromosome, start, stop, reference, variant) with CIViC annotations (average, 6.7 variants per sample). The OpenCAP output report for variants observed on original sequencing and validated by the CIViC smMIP capture panel for CRC1 is shown in the Data Supplement. For each identified clinical variant, links to external databases, CIViC variant descriptions, associated CIViC assertions, and associated CIViC evidence items are provided. Associated evidence items provide a brief description of the clinical relevance, links to CIViC evidence items, and associated citations. An illustrative output report that displays most OpenCAP features, including CIViC variant descriptions and CIViC assertions, was created using a previously reported patient from the literature^27^ (Data Supplement).

## DISCUSSION

OpenCAP is a resource for users to develop a custom capture panel that can be easily linked to actively maintained clinical relevance summaries. The methods described by OpenCAP to build a clinical capture panel offer several advantages relative to existing design paradigms. Use of an open-source database provides a systematic mechanism to survey existing literature within precision oncology to identify variants that are relevant for capture. In addition, the public API permits rapid mapping of identified somatic and germline variants to CIViC clinical relevance summaries. Most importantly, the variants covered by CIViC and associated clinical summaries can be updated in real time as knowledge is entered into the database to accommodate new information discovered within the field of precision oncology.

The smMIP capture method for sequencing provides inherent error correction capability, scalability to detect ultrasensitive variation, and cost effectiveness within a modular design. Combining the public access CIViC database with an ultrasensitive and versatile capture reagent provides an advantageous and principled method for building precision oncology capture reagents. This approach could enable a standardized framework for detecting and interpreting cancer-relevant genomic variation, lowering barriers to use of genomic analysis in the clinical practice of oncology. For maximal flexibility, OpenCAP describes methods for using both unique molecular identifiers (UMIs) and non–UMI-based probes to capture variants of interest.

The CIViC smMIP capture panel used Variant Evidence Scores and SOIDs to identify variants of interest for targeting. However, alternate filtering strategies have been outlined in OpenCAP documents. Regardless of variants targeted for capture, the presented research helped to show that CIViC variants and variant coordinates can be used for accurate capture panel design (95% detection accuracy with Pearson’s *r* = 0.885 for VAFs). This finding helps to validate that the methods described in OpenCAP can be used to accurately interrogate desired variants of interest.

Like all targeted reagents, the preliminary CIViC smMIP design has limitations that can be addressed with future iterations. First, the reagent design is limited by the current knowledge within CIViC. Extensive curation from certain groups (eg, the University Health Network curation of *VHL* variants) disproportionately increases representation for certain genes, cancers, and variant types. Conversely, lack of curation in certain areas shows a disproportionate decreased representation. To address existing curation disparities, CIVIC has joined the Variant Interpretation for Cancer Consortium (VICC)^28^ to integrate multiple variant interpretation knowledgebases into a single meta-knowledgebase. Successful execution of the aims outlined by the VICC would result in harmonization of information from CIViC, the Cancer Genome Interpreter,^29^ Clinical Knowledgebase,^30^ MolecularMatch, OncoKB,^16^ Precision Medicine Knowledgebase,^31^ and others. This would allow users to leverage variant interpretations across multiple platforms for building custom capture panels that are linked to clinical relevance summaries.

In summary, the methods described here validate that community curated data on clinically relevant cancer variants can provide a systematic and dynamic method for capture reagent design. The curated coordinates in the database accurately map to desired variants, and probes designed using these coordinates show accurate recapitulation of the genomic landscape described by orthogonal sequencing. It is our hope that OpenCAP will provide the research community with a novel method to develop next-generation sequencing–based oncology panels.

## APPENDIX

### Determining Eligible Clinical Interpretations of Variants in Cancer Database Variants for Single-Molecule Molecular Inversion Probe Capture

#### Filtering based on the Variant Evidence Score.

All variants within the Clinical Interpretations of Variants in Cancer (CIViC) database are built on evidence statements that have been manually curated from the medical literature. Given that variants within the CIViC database have diverse quantity and quality of evidence support, the Variant Evidence Score was developed to calculate the relative abundance of total available curated evidence for each variant. The Variant Evidence Score reflects the strength of the evidence that was curated and the total amount of curation that has been completed for each variant. To determine evidence strength, the Evidence Level Score and the Trust Rating Score were calculated. The Evidence Level Score is a 10-point scale that weighs the evidence strength based on category. Broadly, highest points are awarded to large clinical studies, and lower points are awarded to case studies, in vitro studies, and inferential evidence. The Trust Rating Score is a 5-star scale that reflects the curator’s confidence in the quality of the study. To determine the total level of curation for each variant, Evidence Level Scores were multiplied by Trust Rating Scores and summed across all evidence items. This final value (ie, the CIViC Variant Evidence Score) was incorporated into the CIViC database and is now available for all variants in the CIVIC Web interface, regular data releases, and application programming interface. Using the CIViC Variant Evidence Score, variants within the top 10% of total curation (corresponding to a Variant Evidence Score > 20 points) were selected to develop the CIViC single-molecule molecular inversion probe (smMIP) capture panel and were eligible for smMIP targeting. Of note, the CIViC Variant Evidence Score evaluates the total level of curation within the database and does not reflect the community consensus of clinical relevance. In addition, the CIViC Variant Evidence Score does not differentiate from conflicting or confounding evidence and weights all evidence based on the algorithm described earlier.

#### Filtering based on the sequence ontology identification number.

Variants were also filtered to only include variants that could be analyzed using a DNA-based sequencing platform. This required use of curated sequence ontology identification numbers (SOIDs). Within CIViC, SOIDs are manually classified as DNA based, RNA based, and/or protein based (Data Supplement). For example, variants with the variant type of “missense_variant” would be labeled as “DNA-based,” whereas variants with the variant type of “transcript_variant” would be labeled as “RNA-based.” Variants that had a “DNA-based” SOID were considered eligible for smMIP targeting, and variants whose SOIDs were “RNA-based” and/or “protein-based” were ineligible.

### Categorization of Variants Based on Length

Using CIViC curated coordinates, variant length was determined (ie, variant start position minus variant stop position). This difference inferred the total number of smMIP probes required to adequately assess each variant.

#### Hotspot targeting.

If the variant length was < 250 base pairs, the variant was eligible for hotspot targeting. For variants that required hotspot targeting, smMIP probes were designed for the genomic region indicated in the CIViC database.

#### Sparse exon tiling and full exon tiling.

If the variant was > 250 base pairs, the variant required some or total tiling of the protein coding exons. For all variants that required sparse exon tiling or full exon tiling, the representative transcript from the CIViC database was used to obtain all possible exons associated with each Ensembl gene. The Ensembl gene was used to obtain all possible exons (biomart=“ENSEMBL_MART_ENSEMBL”, host=“grch37.ensembl.org”, dataset=“hsapiens_gene_ensembl”). Exons were further filtered by Biotype to remove untranslated regions. Some large-scale copy number variants (ie, “AMPLIFICATION,” “LOSS,” “DELETION”) were eligible for sparse tiling, wherein 10 probes distributed across the exons of the gene were retained to enable assessment of copy number state. Other variant types such as “MUTATION” or “FRAMESHIFT MUTATION” required tiling of all protein coding exons. Categorization of all variants eligible for capture is described in the Data Supplement. For variants that required full exon tiling, overlapping smMIPs (ie, at least 1 base pair of overlap) were designed to tile across all protein coding exons in the gene that encompassed the variant. For variants that required sparse exon tiling, approximately 10 smMIPs were designed to cover a portion of the transcript.

### smMIP Sequencing and Data Analytics

Sequencing library construction and balancing of the probe pool were performed as described previously,^21^ and sequencing was performed using an Illumina NextSEquation 500 (Illumina, San Diego, CA). Probes were excluded from the final reagent if they demonstrated poor hybridization to target sequence during initial quality checks.

Sequence data analysis was performed as previously described^21^ with 3 enhancements. First, consensus reads were generated using the fgbiotools (http://fulcrumgenomics.github.io/fgbio/) CallMolecularConsensusReads utility with parameters “–error-rate-post-umi=30–min-reads=2–min-input-base-quality=20”. Second, a custom variant caller was used to identify all consensus calls at a site having at least 2 supporting reads with a minimum specified mapping quality (mapping quality score > 0). Third, variants were required to be detected on at least 4 DNA strands (at least 2 positive and at least 2 negative) to be considered real, rather than postbiologic artifacts (Eijkelenboom A, et al: J Mol Diagn 18:851-863, 2016). Collectively, these provisions require that at least 2 reads are derived from a common unique molecular identifier to create a consensus read and that multiple consensus reads in both directions support the apparent variant. This helps to exclude preanalytic artifacts reflecting DNA damage and stochastic errors that occur during library construction and sequencing. DNA input ranged from 100-500 ng across samples; however, any sample with an overlapping variant that had a variant allele frequency (VAF) < 5% used 500 ng to increase the number of template molecules interrogated.

### Orthogonal Sequencing and Data Analytics

Orthogonal sequencing data from previously conducted whole-exome or genome sequencing was used to validate the CIViC smMIP capture design. Sequencing alignment and somatic variant calling for the acute myeloid leukemia (AML) sample AML31 was performed according to Griffith et al.^26^ Briefly, reads were aligned to GRCh37 using Burrows-Wheeler Aligner (BWA) v0.5.9 (Li H, Durbin R: Bioinformatics 25:1754-1760, 2009), and variants were called using 1 of 7 variant callers listed in the article. Sequencing data from the small-cell lung cancer (SCLC) samples, oral squamous cell carcinoma (OSCC) samples, and Hodgkin lymphoma (HL) samples were analyzed using the Genome Modeling System^2^ at the McDonnell Genome Institute. Reads from these studies were aligned to the reference genome (hg19/GRCh37 or hg38/GRCh38) using BWA-MEM v0.7.10 (Li H, https://arxiv.org/abs/1303.3997), and duplicates were marked by Picard (http://broadinstitute.github.io/picard/) and/or SAMBLASTER v0.1.22 (Faust GG, Hall IM: Bioinformatics 30:2503-2505, 2014). For the SCLC samples, single nucleotide variants (SNVs) were called using SomaticSniper (Larson DE, et al: Bioinformatics 28:311-317, 2012; Larson DE, et al: Curr Protoc Bioinformatics 45:15.5.1-8, 2014), VarScan (Koboldt DC, et al: Genome Res 22:568-576, 2012), and Strelka (Saunders CT, et al: Bioinformatics 28:1811-1817, 2012)^12^; small insertions and deletions (indels) were called using GATK (McKenna A, et al: Genome Res 20:1297-1303, 2010), Pindel (Ye K, et al: Bioinformatics 25:2865-2871, 2009), VarScan2 (Reble E, et al: Psychiatr Genet 27:62-70, 2017), and Strelka. For OSCC samples, SNVs were detected using SomaticSniper v1.0.4, VarScan2 v2.3.6, Strelka v1.0.11, SAMtools r982 (Li H, et al: Bioinformatics 25:2078-2079, 2009), and Mutect v1.1.4 (Cibulskis K, et al: Nat Biotechnol 31:213-219, 2013). Small indels were detected by GATK v5336 (https://software.broadinstitute.org/gatk/), VarScan2, Strelka, and Mutect. For HL samples, SNVs were called using the intersection of SomaticSniper v1.0.4, VarScan v2.3.6, Strelka v1.0.11, and Mutect v1.1.4, and indels were called using GATK, Pindel v0.5, VarScan v2.3.6, and Strelka v1.0.11. For these 3 cohorts, variants identified by automated callers were subjected to heuristic filtering (removal of variants with low VAF [< 5%] or low coverage [< 20 times in tumor or normal track]), and false positives were removed via manual somatic variant refinement.^23^ If variant coordinates corresponded to GRCh38, their coordinates were converted to GRCh37 using LiftOver (Hinrichs AS, et al: Nucleic Acids Res 34:D590-D598, 2006). For the colorectal cancer (CRC) cohort, sequencing, variant calling, and clinical annotation were performed according to methods highlighted in Pritchard et al (J Mol Diagn 16:56-67, 2014). Briefly, sequencing was performed using Illumina next-generation sequencing (Illumina, San Diego, CA), and sequencing reads were aligned using BWA v0.6.1 and SAMtools v0.1.18. Indel realignment was then performed using GATK v1.6, and duplicate reads were removed using Picard v1.72. SNV and indel calling was performed using the GATK Universal Genotyper with default parameters and VarScan v2.3.2.

### Assessment of Variants Missed Using the CIViC smMIP Capture Panel

Of the 65 variants identified on exome sequencing, all but 4 were also identified using CIViC smMIP sequencing. One variant was missed as a result of lack of adequate coverage, 2 variants were missed as a result of low-performing probes, and 1 variant was retrospectively considered ineligible as a result of smMIP design. The variant missed as a result of inadequate coverage was a *TP53* (p.G266R) variant identified in the AML31 tumor sample. Original sequencing indicated that this variant was present at 0.04% VAF; therefore, given smMIP coverage of 2,388 reads at this site, there was only a 0.01% chance that this variant would have been detected (1-tailed probability of ≥ 4 reads [K] of 2,388 reads [n]; *P* = .0046). However, this low-prevalence variant could have been recovered given additional sequence coverage. In addition, there were 2 variants missed as a result of low molecular inversion probe (MIP) performance. The first variant that was missed (chr10:g.89690805G>A in the SCLC8 tumor sample at 94% VAF) was a result of poor performance of the MIP covering the region of interest in the reverse direction. This MIP showed only 1 aligned read across all 36 samples and had no aligned reads in SCLC8. Despite the fact that there was extensive support from the forward MIP (95% VAF with 34 of 35 consensus reads), the requirement that both forward and reverse reads show support prevented this variant from being called. The second missed variant (*PTEN* e8-1 in the SCLC4 tumor sample at 100% VAF) was a result of low performance of MIPs in both directions. Even though both the forward and the reverse MIPs showed variant support, the forward MIP only contained 2 consensus reads and the reverse MIP only contained 1 consensus read, preventing it from being called as somatic. The final variant (chr17:g7577094C>T in the CRC5 tumor sample at 32% VAF) was retrospectively considered ineligible because the original smMIPs developed to cover the eligible STK variant called for sparse tiling (ie, identification of copy number change). As such, the variant was contained by a region that did not have full coverage in the forward direction. When evaluating the reverse MIP that contained this site, we observed a 34% VAF (402 of 1,184 reads), which was comparable to the original sequencing data. However, lack of a secondary probe designed against the complementary DNA strand prevented this variant from being called as somatic.

### Code and Accessibility

All raw data, analysis, and preprocessing code, are publicly available on the GitHub repository (https://github.com/griffithlab/civic-panel/). All plots were produced using the MatPlotlib library in Python (Hunter JD: Comput Sci Eng 9:90-95, 2007). The raw sequencing data are publicly available for most projects included in this study (Data Supplement). The smMIP sequence analysis pipeline is accessible on bitbucket (https://bitbucket.org/uwlabmed/smmips_analysis).

### Data Statement

The raw smMIP sequencing data associated with samples from the McDonnell Genome Institute (head and neck squamous cell carcinomas, SCLCs, HLs, and AMLs) have been submitted to the Database of Genotypes and Phenotypes under accession No. phs001890.v1.p1. Institutional review board approval, consent forms and versions, and other demographic data are provided in this submission. The raw smMIP sequencing data associated with samples from Washington University (CRCs) have been submitted to the Sequence Read Archive under accession No. PRJNA529857.
